# New York Academy of Medicine

**Published:** 1867-02

**Authors:** 


### NEW YORK ACADEMY OF MEDICINE.

Dr. Post again introduced Dr. Quinby's patient for the purpose of showing the progress of the case. He remarked that there could here be no arrest to the growth of the leg, since no portion of the tibia or fibula had been removed, and that, as a result of this operation, the patient was not prevented from wearing a boot. The patient then exhibited his control over his movements by walking in different gaits, jumping, etc.

### Continuation of the Discussion on Chronic Metritis.

Dr. Kammerer remarked that the discussion of chronic metritis had gone back to the very premises—to wit: whether there was such a disease as chronic metritis or not. He thought that the cause of this variance lay in the fact that pathological anatomy had outstripped clinical observation. He would ask, in considering this topic, whether or not this derangement in nutrition was marked by exudation, a stagnation of the circulation, etc. Hyperæmia was no symptom, since the sexual organs suffered a recurrence of that condition every four weeks. Irritation should certainly not be confounded with inflammation; and regarding Virchow's explanation of the phenomena of inflammation as decidely the best, he found none willing to claim that the conditions as laid down by that eminent authority were here present. That they were met with in perimetritis with pelvic peritonitis, and in endometritis, he had no doubt; but as far as the body was concerned, he should be more reserved in his opinion. French authors have no doubts of the existence of metritis. Prof. Klob, of Vienna, whose work, published in 1863, has not yet been translated, who made 1000 autopsies in the General Hospitals, failed to find a single case of genuine metritis. All the changes pointed only to hyperæmia. Entertaining these views, backed as they were by authority so eminent, he could only regard the term chronic metritis, as a misnomer. The subjective symptoms of chronic metritis, then, if we may allow the term as a matter of convenience, so far as our clinical observation goes, are derangements of the functions, amenorrhœa, dysmenorrhœa, sterility, etc, while the objective are an enlarged organ, with ulcerations, abrasions, and discolorations. We may very readly make out the size by what is technically known as the double touch, *i. e.* by the use of the sound simultaneously with palpation of the abdomen. The cavity may be found deeper and wider, the organ itself enlarged, which latter is, after all, the only pathognomonic symptom. He would admit, however, that we must not expect to be invariably fortunate in making out the enlagement of the organ, since the thickness of the tegumentary coverings has quite frequently foiled us. He also did not regard a tender as neccessarily an inflamed uterus—for a certain amount of hyperæsthesia might be present, without looking towards any very grave disease, since pain of any account would indicate peritonitis or ovaritis. The pathological anatomy of the disease will be found to reveal a morbid condition rather than a morbid process. We may find the muscular fibres hypertrophied and the connective tissue increased in quantity, but even these may constitute merely a quantitative anomaly, and that, too, within the limits of health. The chronic induration resulting from engorgement was due to an increase of the connective tissue at the expense of the muscular fibres; the organ may have an anæmic hue, and may creak under the knife. All this was not inflammation, but merely the result of long-continued congestion or hyperæmia.

He might refer to the two forms of hyperæmia as proper to be borne in mind, while considering the case now in dispute. The arterial form is well typified in menstruation and puerperal fever; while venous congestion is exemplified in those instances where obstacles are offered to the reflux of blood, as in displaçements, flexions, adhesions, and the embedment of fibrous tumors within the walls. He conceived that the diverse opinions formed regarding this condition of things might be explained by the fact that not enough stress had been laid upon such complications as peritonitis or its sequelæ of adhesions, latero-versions by the contractions of ligaments, etc. Chronic peritonitis was likewise a frequent complication.

The best remedies within our grasp were antiphlogisties and frequent tepid baths. For the accompanying endometritis, the very first condition was to keep the orifice well dilated for the escape of the secretions, by which the mucous membranes are relieved, and the general condition rendered more comfortable. Thorough dilatation might be effected through the well known agency of bougies and sea-tangle (*laminaria digitata*) or sponge tents. He desired to call attention to two local remedies, which had not yet come into general use. The first of these was *pyroligneous acid*, first introduced to the profession by Dr. Meyer, of Berlin, and which was held in high esteem, especially by German practitioners. He would assure those disposed to give this agent a trial, that there was no danger in its use, provided the article were pure. The second remedy was *carbolic acid* in concentrated solution, which might be introduced into the cavity by a brush. No bad results have been known to occur; the only obstacle to its application he knew of, was the presence of acute peritoneal irritation or sub-acute inflammation.

When displacements existed, our only resort was, of course, to adjust them if possible; but all cases of slight hypertrophy he would let alone, unless, perhaps, when the cervix assumed the form of prolapse, he might attack the neck by surgical means, such as by the écraseur or by the electro-caustic method.

When the hyperæmia was due to a general cause,—to cardiac complications or tuberculosis, but little mitigation of the patient's sufferings need be expected. After all, the only real cure, in his opinion, was effected when senile atrophy ended the woman's parturient history.

Dr. Peaslee said: He had hoped to have listened instead of speaking to-night; but some of the topics which had been discussed, he thought required further elucidation.

He thought that the view he had taken at the previous meeting, of the subject under consideration, was both logical and scientific, and one which could not be successfully opposed. He had without argument admitted the existence of parenchymatous inflammation of the uterus; though he was aware that Bernutz and others doubt if acute parenchymatous inflammation of the unimpregnated uterus ever occurs; and Klob, as Dr. Kammerer has told us, admits that he has never, in all his *post mortem* examinations, been able to demonstrate parenchymatous metritis in any form. Neither had he (Dr. P.) discussed the nature of inflammation, since that would have led him too far from his main object. Dr. Kammerer has stated that its most important characteristic is an exudation of the plasma of the blood between the histological elements of the part affected, as all must admit in the present state of general pathology. Dr. P. had simply specified the symptoms recognised by all writers as indicative of inflammation of the parenchyma of the uterus, and admitting its existence where they do exist, he had refused to admit it where they do not. But he had shown that these symptoms do not exist, and have not existed at all, in a large majority of all the cases generally included under the term chronic parenchymatous metritis; while they do appear, and then disappear again to recur, in the minority of these cases. He therefore decided that the majority are not cases of metritis at all, while metritis recurred from time to time in the minority of the cases. On further examination he found the former presented the symptoms of chronic congestion merely, while the latter presented the same state with attacks of metritis supervening upon it.

This view had been somewhat earnestly criticised at the last meeting, as he (Dr. P.) hoped it might, since it had been held by him the last fifteen years, and finds constant confirmation in his practice, and he regretted that Dr. Barker could not be present this evening, since he might wish to reply to criticisms which he (Dr. P.) should in turn feel obliged to make.

Alluding to Dr. Barker's division of the cases of chronic metritis into three classes, Dr. P. said he (Dr. B.) had committed the too common error of confounding the effects, or sequelæ, of inflammation with inflammation itself. Dr. Churchill has adopted the same classification (and added two or three classes more); but he so blends acute and chronic metritis in his account of parenchymatous inflammation of the uterus, that one can get no clear idea of the difference between them on the one hand, or between them and induration, or softening, or suppuration on the other. Dr. P. would speak only of the two first classes of cases recognised by Dr. Barker, viz.: (1) chronic metritis complicated with indurations; and (2) the same complicated with softening.

Twenty-five years ago (said Dr. P.), it was an axiom in general pathology that chronic inflammation produces induration or hardening, while acute inflammation produces softening. This statement, however, does not present the facts as they really exist, but merely a simple view of them. In the first place, we must remember that the characteristic element of the inflammatory process is an exudation of the plasma of the blood from the capillary vessels of the part affected into the intercapillary spaces, and among its histological elements. As this is, of course, itself softer than the tissue proper, the whole mass of tissues and plasma thus become softer than the tissues above in their natural state. An immediate effect, then, of inflammation in all cases (acute or chronic) is softening. But this exudation among the tissues may subsequently be disposed of in either of three entirely distinct ways. It may (1) be entirely and promptly reabsorbed, in which case it is customary to say that the inflammation terminates in resolution; or (2) it may become organized, and thus be permanently blended with the tissues of the part, and in this case the whole mass of tissue and neoplasma together, will (in the case of a soft solid) be after a time harder than before, and we say *induration* has taken place; or (3) the exuded plasma may degenerate into pus, and be thrown out from the part, when we say *suppuration* has ensued. The accurate statement of the facts is, therefore, this: inflammation directly produces softening, as the result of its exudation of the blood plasma into the part; and if hardening ensues after a time in a part where inflammation had existed, it is due to an organization of the same plasma which at first rendered the same part softer. It does not depend on the character of the inflammation, whether acute or chronic, but merely on the fact of the organization of the plasma, instead of its reabsorption or its degeneration; and this organization requires a certain amount of time. In other words, it is a *chronic result* of an inflammation, though the latter may have been itself acute or chronic. Metritis, therefore, complicated with softening, is simply actue metritis; for the softening is one of its essential and never absent results. Of course we do not here consider fatty degeneration and other forms of softening of the uterine tissue, which have no known or suspected relation to the inflammatory process. And chronic metritis, complicated with induration, is simply hardening of the uterus from the organization of the product of a previous inflammation. Congestion is, however, very liable to continue for a longer or shorter time after the inflammatory process subsides, and hence we often find chronic congestion coëxisting with induration. Besides, we may have recurrent inflammations in an indurated uterus; but, if so, this inflammation needs no peculiar treatment on account of the induration; and the latter still remains to be treated after the inflammation subsides. The distinction of the two classes referred to, therefore, of complications with chronic parenchymatous metritis, are pathologically incorrect, and besides have no therapeutical value. On the other hand, we remember that induration is continually found without any inflammation or even congestion of the part; though sub-involution after parturtion is sometimes mistaken for it.

Another error which Dr. P. had to specify was the confounding of engorgement with inflammation in the case of any organ. Some, however, go further than this, and use the terms congestion, engorgement, and inflammation, as if they mean precisely the same thing. When we study the phenomena of inflammation artifically produced in the web of the frog's foot, or the wing of the bat, we find that the stimulant which we apply produces, 1st, an increased flow of blood to, and through, the capillaries of the part (local determination of blood). 2d, a loss of power of the vessels from over distension, and, therefore, a diminished flow of blood through them (congestion); and 3d, an entire stagnation of the blood in the capillaries, and the exudation of its plasma into the inter-capillary spaces, while the corpuscles remain in the vessels (inflammation.) On the other hand, when recovery takes place, the inflammation first fades into congestion, and this into the healthy condition of the part. The difference, therefore, between inflammation and congestion is distinctly marked, and always demonstrable under the microscope. But the term engorgement simply means that the part is choked up with an extra amount of fluid, either in the vessels or between them, or both. Hence it may in one case mean mere congestion, and in another, the state produced by inflammation (the vessels distended with blood corpuscles and the plasma exuded from the vessels). But it is never properly applied to the inflammatory process itself. It has, therefore, no scientific value; and the sooner it is entirely dropped from the nomenclature of pathology, the better.

Another point to which Dr. P. desired to call attention is the statement, which had of late become quite fashionable, that the body and the cervix of the uterus are distinct organs, which he characterized as entirely incorrect, whether we consider their relations in a developemntal, a histiological, an anatomical, a physiological, a pathological, or a therapeutical point of view.

1st. *Developmentally*, the cervix and the body are one and the same organ. The internal female genital organs are developed from the ovaries; each developing one-half of the uterus and one Fallopian tube. The body and cervix are continuous in structure from the first. At birth, however, the cervix is nearly twice as large as the body, constituting about two-thirds of the whole organ; and the uterus has the form of a cone, with its apex at the fundus.

2d. *Histologically*, the body and the cervix uteri are the same also. There are the same tissues in both, and both are supplied with vessels and nerves from the same sources. The arrangment of the mucous lining of the cervix is different from that of the body; but the tissue is the same. And besides the mucous membrane, the nerves, bloodvessels, and lymphatics, and the pertioneal investment, there is nothing but collagenous (or connective) tissue and non-striated fibre in either.

Dr. Barker has spoken of a similarity of the uterus, in a histological point of view, to cartilage and bone. But cartilage has neither bloodvessels, lymphatics, nerves, nor muscular fibre, nor any other of the tissues found in the uterus. It has only two histological elements, cartilage cells and a hyaline substance, in which they are imbedded. Bone, on the other hand, has no muscular fibres or collagenous tissue like the uterus; but owes its peculiarities to its phosphate of lime and other salts combined with osteine. Histologically, therefore, the comparison of the uterus with cartilage and bone, is incorrect.

3d. *Anatomically* considered, the cervix and the body are but parts of the same organ. It has been stated that the muscular fibres of the body of the uterus are nowhere prolonged into the neck except posteriorly. If this were really so, the cervix would be torn from the body at their junction in front in case of parturition with rigidity of the cervix; and the child would be expelled into the peritonealy cavity through the rupture. But what are the anatomical facts? Simply that the longitudinal fibres are prolonged from the body of the uterus into the cervix both anteriorly and posteriorly. Dr. P. published this statement in his work on histology, ten years ago; and he who dissects the uterus of a woman who dies within a week or two after parturition, can easily verify it. As to the arrangement of muscular fibres, there is a striking analogy between the uterine body and neck, and the body and neck of the bladder; and neither of these organs could expel its contents with the precise arrangement that obtains.

4th. *Physiologically*, a distinction is to be made between the body and the neck of the uterus; but even here they are to be regarded as one and the same organ. The function of menstruation is performed by the body, and not at all by the cervix, but the Fallopian tubes also participate with the body in that function. Gestation also is accomplished by the body, and very slightly, if at all, by the cervix (for he would not here discuss the question of the obliteration of the cervix by merging into the body in the latter months of pregnancy). The mucus secreted by the cervix differs from that of the body also. But the great culminating function of the body of the uterus is *par-*

*turition*, and in this the cervix acts an important part—as a portion of the same organ. Viewed in respect to this function, the cervix is merely the sphincter muscle of the body, as at the end of the rectum we find the sphincter muscle of that canal. The office of both is also the same, viz., to prevent the premature evacuation of the canal which it closes. But a sphincter can never open itself; it must be forced open, as is the case with the sphincter ani, or pulled and forced at the same time, as is the case with the cervix uteri and the neck of the bladder. And the continuation of the longitudinal fibres of the body of the uterus into the cervix, both anteriorly and posteriorly, as has been explained, is the only arrangement which could accomplish this double effect on the cervix which has just been mentioned. The cervix and the body of the uterus are, then, not distinct organs.

5th. Nor do we discover the asserted independence of the cervix uteri and the body, when we consider their *pathological* relations. Endocervicitis may, and often does, exist without extending into the uterine cavity, and becoming endometritis also; and cervicitis occurs independently of metritis proper; though both metritis and endometritis far less frequently occur uncomplicated with cervicitis and endocervicitis. Still, we know that an irritation in the cervical canal by a sponge-tent frequently produces endometritis as well as a miscarriage in case of pregnancy; and that the common cause of metritis proper in newly-married woman is contusion of the cervix. In other words, certain pathological states of the cervix often extend their influence directly to the body of the uterus, as we might expect; and others extend more frequently in the reverse direction: though both these facts are inconsistent with the idea that these parts are distinct organs.

6th. Finally, the cervix and the body of the uterus are not distinct organs in a *therapeutical* point of view; and this is a fact of the highest practical importance. If we cannot affect the body of the womb, as has been asserted, by any application to the cervix, of course all local treatment must be abandoned as useless; since applications to any other part must, by parity of reasoning, be even less efficient still. If the abstraction of blood from the cervix does not modify the circulation of the body of the uterus, when both are supplied by vessels from the same source, he could not imagine how we could affect the uterine circulation by leeching or scarification at any other point. But what are the facts? He had noticed that scarifications, often repeated, of the cervix, in the cases under discussion, produce immediate relief in most cases, and which is frequently permanent; and Dr. Barker also has admitted the relief, though he believes it is merely temporary. But any degree of relief shows that the circulation of the body of the uterus is thus modified. Besides, Dr. Barker places his main confidence, so far as local treatment is concerned, in vaginal injections of warm water—a practice quite irrational if the cervix and body are independent organs; but which also was occasionally resorted to by himself, on the principle that the water thus applied to the cervix extends its effects to the body also.

But he (Dr. P.) would not extend his remarks further on the question of the dependence of these two portions of the uterus. Dr. Kammerer had remarked that he knew of no treatment which would remove the induration of the uterus before mentioned. Dr. P. regretted that he could not shed much light on that point; but he thought we must trust mainly to the bichloride of mercury, bromide of potassium, electro-magnetism, and especially to *time.*

The meeting then adjourned.

